# Sex differences in the therapeutic effect of unaltered versus NFκB sensing IL-4 over-expressing mesenchymal stromal cells in a murine model of chronic inflammatory bone loss

**DOI:** 10.3389/fbioe.2022.962114

**Published:** 2022-08-15

**Authors:** Huaishuang Shen, Junichi Kushioka, Masakazu Toya, Takeshi Utsunomiya, Hirohito Hirata, Ejun Elijah Huang, Masanori Tsubosaka, Qi Gao, Xueping Li, Victoria Teissier, Ning Zhang, Stuart B. Goodman

**Affiliations:** ^1^ Department of Orthopaedic Surgery, Stanford University School of Medicine, Stanford, CA, United States; ^2^ Department of Orthopaedic Surgery, Kyushu University, Fukuoka, Japan; ^3^ Department of Bioengineering, Stanford University, Stanford, CA, United States

**Keywords:** sex differences, chronic inflammatory bone loss, wear particles, mesenchymal stromal cell, macrophage

## Abstract

Wear particles from joint arthroplasties induce chronic inflammation associated with prolonged upregulation of nuclear factor kappa-B (NF-κB) signaling in macrophages and osteoclasts, which leads to osteolysis and implant loosening. Mesenchymal stromal cell (MSC)-based therapy showed great potential for immunomodulation and mitigation of osteolysis *in vivo*, especially in the chronic phase of inflammation. We previously generated genetically modified MSCs that secrete the anti-inflammatory cytokine interleukin 4 (IL-4) in response to NF-κB activation (NFκB-IL-4 MSCs). However, whether the impact of sexual difference in the internal environment can alter the therapeutic effects of IL-4 over-secreting MSCs that simultaneously mitigate prolonged inflammation and enhance bone formation remains unknown. This study investigated the therapeutic effects of unaltered MSCs versus NFκB-IL-4 MSCs in mitigating chronic inflammation and enhancing bone formation in male and female mice. The murine model was established by continuous infusion of polyethylene particles contaminated with lipopolysaccharide (cPE) into the medullary cavity of the distal femur for 6 weeks to induce chronic inflammation. Unaltered MSCs or NFκB-IL-4 MSCs were infused into the femoral intramedullary cavity in sex-matched groups beginning 3 weeks after primary surgery. Femurs were harvested at 6 weeks, and bone marrow density was measured with micro-computational tomography. Numbers of osteoclast-like cells, osteoblasts, and macrophages were evaluated with histochemical and immunofluorescence staining. cPE infusion resulted in severe bone loss at the surgery site, increased tartrate-resistant acid phosphatase positive osteoclasts and M1 pro-inflammatory macrophages, and decreased alkaline phosphatase expression. MSC-based therapy effectively decreased local bone loss and polarized M1 macrophages into an M2 anti-inflammatory phenotype. In females, unaltered MSCs demonstrated a larger impact in enhancing the osteogenesis, but they demonstrated similar anti-inflammatory effects compared to NFκB-IL-4 MSCs. These results demonstrated that local inflammatory bone loss can be effectively modulated via MSC-based treatments in a sexually dimorphic manner, which could be an efficacious therapeutic strategy for treatment of periprosthetic osteolysis in both genders.

## Introduction

Total joint arthroplasty is an effective therapeutic option for patients with end-stage degenerative arthritis (Goodman, 2007; Abdelaal et al., 2020). Although follow up studies showed improvements in pain and functional outcome, wear particle-induced periprosthetic osteolysis (PPOL) and further aseptic loosening are the major long-term complications that lead to revision surgeries with higher complication rates and great economic burden (Kurtz et al., 2014; Price et al., 2018). To prevent this debilitating problem, novel interventions to reduce chronic inflammation and bone resorption in the early phase are highly desirable.

Chronic low-grade inflammation exhibits strong correlation with PPOL (Gallo et al., 2013a; Gallo et al., 2013b; Goodman and Gallo, 2019). In the presence of wear particles, the periprosthetic microenvironment experiences a macrophage-mediated chronic inflammatory reaction with persistent release of pro-inflammatory cytokines, chemokines, and other molecules, via prolonged upregulation of transcription factors such as nuclear factor κB (NF-κB) (Xu et al., 2009; Abu-Amer, 2013; Goodman et al., 2019; Goodman and Gallo, 2019).

We previously demonstrated that MSC-based therapy exhibits great potential for immunomodulation and enhancing osteogenesis *in vitro* and *in vivo*, in a microenvironment of acute or chronic inflammation (Utsunomiya et al., 2021b). To enhance bone formation and modulate inflammation simultaneously, we generated genetically modified MSCs that over express interleukin 4 (IL-4) in response to NF-κB activation by lipopolysaccharide (LPS) contaminated particles (cPE) (Lin et al., 2017; Lin et al., 2018). Furthermore, local delivery of genetically modified MSCs in a murine bone loss model attenuated the bone resorption but showed equally effective effects as treatment with unaltered MSCs (Zhang et al., 2021b). Next, prior preclinical studies demonstrated promising effects of different pharmacological interventions on decreasing bone resorption due to wear particles (Goodman et al., 2005; Smith and Schwarz, 2014); however, clinical trials have been less successful. For example, anti-TNF agents were not shown to be efficacious in a clinical trial for patients with particle-associated periprosthetic osteolysis (Schwarz et al., 2003). Bisphosphonate therapy was less effective in treating inflammation-induced osteolysis than generalized osteoporosis (Schwarz et al., 2000); further long-term follow up studies are necessary regarding compliance issues and potential adverse effects. Overall, the non-operative treatment of particle-induced chronic inflammation and periprosthetic osteolysis remained an unsolved problem (Schwarz, 2008; Goodman et al., 2014). Further, interestingly, sex was not included in most preclinical studies, which now needs additional consideration as an important variable (Clayton and Collins, 2014; Sandberg et al., 2015). Hormonal effects associated with sex may modulate the responses of the innate immune system and in inflammatory conditions such as particle-induced periprosthetic osteolysis (Laurent et al., 2014). Thus, the therapeutic effects of MSC-based therapy may be sexually dimorphic and were not investigated previously in preclinical studies of chronic inflammatory bone loss. Whether IL-4 secreting MSCs can mitigate chronic inflammation and enhance MSC-mediated bone formation effectively in both males and females remains unknown. This study investigated the sex differences in therapeutic effects of unaltered MSCs versus NFκB-IL-4 MSCs in modulating chronic inflammation and osteolysis using murine models in male and female mice.

## Materials and methods

### Isolation of mesenchymal stromal cells

The experimental design was approved by the Institutional Administration Panel for Laboratory Animal Care (APLAC) at Stanford University. We used 10–12 weeks old BALB/c male and female mice, and bone marrow derived MSCs were isolated as previously described (Soleimani and Nadri, 2009; Lin et al., 2018). Briefly, bone marrow was collected from the femur and tibia of males and females and then filtered through a 70-mm strainer. Cells were isolated by centrifugation and resuspended in 15-cm dish with alpha-minimal essential medium (a-MEM, Thermo Fisher Scientific, Waltham, MA, United States) supplied with 10% MSC certified fetal bovine serum (FBS, Invitrogen, Carlsbad, CA, United States) and antibiotic antimycotic solution (100 units of penicillin, 100 μg of streptomycin and 0.25 μg of Amphotericin B per mL; Hyclone, Thermo Scientific). Next, to remove the unattached cells, fresh medium was replaced the next day (passage 1). The cells were allowed to grow to confluence around 3 weeks later. The cells were washed with PBS, detached with trypsin, and then flushed with media into a centrifuge tube and spun down at 400 *g* for 5 min. The cells were detached with trypsin after growing to confluence after 3 weeks, resuspended, and plated at a density of 4,000 cells/cm^2^. This procedure for subculture was repeated twice until pure MSCs were isolated using the murine mesenchymal stromal cell enrichment kit (STEMCELL Technologies, Vancouver, Canada) in passage 6. The cellular morphology was observed under a microscope (Axio Observer 3.1, Zeiss, Oberkochen, Germany). At passage 5, the immunophenotype of the isolated MSCs (CD105^+^/CD73^+^/CD90.2^+^/CD44^+^/Sca1^+^/CD45^−^/CD34^−^/CD11b^−^) was characterized by LSR II flow cytometer (BD Bioscience).

### Generation of NF-κB sensing IL-4 secreting mesenchymal stromal cells

As previously described, the lentiviral vector preparation was conducted (Lin et al., 2017; Zhang et al., 2021a). Briefly, we utilized human embryonic kidney 293T cells (ATCC, Manassas, VA, United States) to transfect the control lentivirus vector the mouse IL-4 secreting pCDH-NF-κB-mIL-4-copGFP expressing lentivirus vector combined with psPAX2 packaging vector and pMD2G VSV-G envelope vector, with the calcium phosphate transfection kit (Clontech, Mountain View, CA, United States) and 25 mM chloroquine. Next, the diluted virus was added in MSC culture medium supplemented with 6 mg/ml of polybrene (Sigma-Aldrich, St. Louis, MO, United States) and infected to murine MSCs at multiplicity of infection (MOI) = 100. The genetically modified cells were GPF positive confirmed by fluorescence microscope after 3 days of infection (BZ-X810, Keyence, IL, United States).

### Enzyme-linked immunosorbent assay and PrestoBlue assay

IL-4 expression by unaltered MSCs and NF-κB sensing IL-4 MSCs with LPS challenge was measured. Cells were seeded in 24-well plates at a concentration of 1 × 10^4^/well, with supplemented culture medium until 80% confluency. Fresh medium was then changed, and cells were cultured with 1 μg/ml LPS (Sigma-Aldrich, St. Louis, MO) or left untreated for 24 h. Supernatant was collected for Enzyme-Linked Immunosorbent Assay (ELISA kit for mouse IL-4, R&D system, Minneapolis, MN, United States). The optical densities were determined using SpectraMax M2e Microplate Readers (Molecular Devices, San Jose, CA, United States) set at 450 nm with wavelength correction set to 540 nm. Cell viability was assessed with PrestoBlue assay (Xu et al., 2015). After collection of supernatant, 1 ml of PrestoBlue solution (10% in medium without serum and phenol red, Invitrogen, Carlsbad, CA, United States) was added to each well, and the plates were incubated at 37°C for 30 min. After incubation, 100 μL of the PrestoBlue solution from each well was transferred to a new well in 96-well plate, and the fluorescence of the test reagent was measured with the excitation/emission wavelengths set at 560/590 nm.

### Ultra-high molecular weight polyethylene particles and preparation of osmotic pumps

As previously described, polyethylene particles were prepared (Lin et al., 2018). Briefly, Ceridust 3610 polyethylene particles (Clariant Corporation, CA, United States) were washed with 100% ethanol and then filtered through a 20 μm pore membrane to exclude larger particles. The filtered particle size was measured as 4.62 ± 3.76 μm with a scanning electron microscope (Zeiss Sigma FESEM, Zeiss Sigma, CA, United States) in the Cell Sciences Imaging Facility at Stanford University. After being dried with a vacuum for 3 days, particles were resuspended in PBS containing 5% Bovine Serum Albumin (BSA, Thermo Fisher Scientific). According to our previous study (Utsunomiya et al., 2021a), the approximate concentration is 3.1 × 10^10^ particles/ml, and the sterility was confirmed by Limulus Amebocyte Lysate assay (Lonza, Portsmouth, NH).

Contaminated polyethylene particles (cPE) were prepared by mixing the particles with 10% BSA/PBS (15 mg/ml) added with 10 ng/ml of LPS (Sigma-Aldrich St Louis, MO) (Greenfield et al., 2010), and then, cPE was loaded into an Alzet mini osmotic pump with an average working rate of 0.15 ml/h (Model 2006; DURECT corporation, Cupertino, CA, United States). All the pumps were incubated in 37°C for 3 days before implantation with connected vinyl catheters and hollow titanium rods.

### Mouse model of bone resorption and chronic inflammation due to contaminated polyethylene particles

**FIGURE 1:**
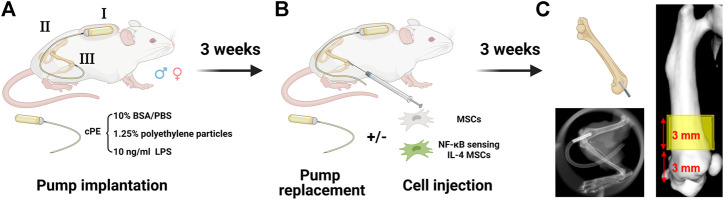
Mouse model and experimental design of chronic inflammation and bone resorption due to contaminated polyethylene particles (created with Biorender.com). **(A)** the murine continuous femoral particle infusion model. I: cPE were continuously infused from a dorsally implanted pump; II: vinyl catheter connecting tube; III: 6-mm titanium hollow rod placed into the distal femur. **(B)** mesenchymal stromal cell (MSC) or NFκB-IL4 MSC injection and pump replacement at the second surgery at 3 weeks **(C)** femurs were harvested at 6 weeks for further analysis. The region of interest (ROI) was defined as a 4 mm × 4 mm × 3 mm box and started 3 mm from distal femur end.

Male and female BALB/cByJ mice (8–12 weeks old) were used for the continuous femoral intramedullary polyethylene particle infusion model as previously described (Figure 1A) (Ma et al., 2009; Lin et al., 2016; Pajarinen et al., 2017). Briefly, the mouse was administered with preoperative analgesia (0.1 mg/kg of buprenorphine, subcutaneously) and inhalation anesthesia (2% isoflurane in 100% oxygen, 1 L/min) on a warm animal surgery station. A lateral parapatellar incision was performed on the right knee joint, and the intercondylar notch into the medullary cavity was created with a series of needles (from 25 to 21 gauges) sequentially. A 6-mm-long hollow titanium rod (23 gauge) was implanted into the distal canal of femur. Then, the titanium hollow rod (Figure 1AIII) was connected to the subcutaneously and dorsally implanted osmotic pump (Figure 1AI) *via* a vinyl catheter tubing (Figure 1AII). To induce chronic bone inflammation, cPE were continuously infused from the pump (Figure 1AI) into the medullary cavity of the distal femur (Utsunomiya et al., 2021a). Three groups were found in both sexes for the following treatments: cPE only; cPE + MSCs; cPE + NFκB IL-4 MSCs. At 3 weeks, the pump was replaced with a new one in each group, and MSCs or NFκB-IL-4 over-expressing MSCs were injected into the medullary cavity through the rod for treatment (Figure 1B). Skin incisions were closed with 5–0 Ethilon sutures. At 6 weeks, mice were euthanized by exposure to CO_2_ followed by cervical dislocation, and femurs were harvested for analysis.

### Micro-computational tomography

After detaching the pumps and rods, μCT scans were performed using TriFoil Imaging CT120 (TriFoil Imaging, Chatsworth, CA, United States) with 49 μm resolution. A three-dimensional (3D) region of interest (ROI) was created, 4 mm × 4 mm × 3 mm, within the distal femur which began 3 mm from the distal end of the femur and proceeded proximally (Figure 1C) (Lin et al., 2016). Next, the bone mineral density (BMD, mg/cm^3^) was analyzed by GEMS MicroView software (threshold: 700 HU).

### Tissue processing for frozen sections

Further, previously harvested and scanned femurs were fixed in 4% paraformaldehyde overnight and decalcified in 0.5 Methylenediamine-tetra-acetic acid (EDTA, pH 7.4) for 2 weeks. After dehydration with 30% Sucrose for 3 days, the specimens were placed in optimal cutting temperature compounds (Tissue PlusR, Fisher HealthCare, Hampton, NH) for frozen specimens. Then, for subsequent histological examination, the ROI located 3 mm from the distal end of femur was cut into 10-µm-thick transverse sections (Lin et al., 2016).

### Osteoblast activity and osteoclast-like cells detection

Osteoblast activity was assessed by alkaline phosphatase (ALP) staining with 1-step NBT/BCIP substrate solution (Thermo Fisher Scientific Rockford, IL, United States). The ALP positive area based on the entire area of the scaffold was calculated using the Qupath image analysis software (Bankhead et al., 2017). Osteoclast-like cells were stained using a leukocyte tartrate-resistant acid phosphatase (TRAP) staining kit (Sigma-Aldrich, St. Louis, MO, United States). Multinucleated TRAP positive cells located on the bone perimeter within the resorption lacunae were defined as osteoclast-like cells. Cell numbers in three randomly selected areas per each section were manually counted according to our previous protocol (Utsunomiya et al., 2021a). The color threshold of each parameter was determined by consensus of three investigators. Also, double-blinded quantitative analysis was conducted by two investigators.

### Immunofluorescent for macrophage polarization analysis

For macrophage detection, the specimens were blocked and permeabilized by 5% BSA with 0.3% Triton X-100 buffer for 60 min at room temperature, followed by primary and secondary antibody incubation. Macrophages/monocytes were identified with rat anti-CD11b antibody (Abcam, Cambridge, MA, United States) followed by Alexa Fluor^®^ 647 conjugated donkey anti-rat IgG (Abcam, Cambridge, MA, United States). M1 pro-inflammatory macrophages were identified with a mouse anti-inducible nitric oxide synthase (iNOS) antibody (Abcam, Cambridge, MA, United States) followed by Alexa Fluor^®^ 555 conjugated donkey anti-mouse IgG (Invitrogen, CA, United States). Next, M2 anti-inflammatory macrophages were identified by rabbit anti-liver arginase (Arg1) antibody (Abcam, Cambridge, MA, United States) followed by Alexa Fluor^®^ 488 conjugated donkey anti-rabbit IgG (Invitrogen, Carlsbad, CA, United States). Slides were mounted by prolong diamond antifade mount with DAPI (Invitrogen, Carlsbad, CA, United States) and imaged using a fluorescence microscope with ×200 magnification (BZ-×810, Keyence, IL, United States). Positive cells in all sections were counted in three randomly selected areas. Cell counting was analyzed with Qupath image analysis software. Double-blinded quantitative analysis was conducted by two investigators.

### Statistical analysis

Statistical analyses were conducted using GraphPad Prism 9 (GraphPad Software, San Diego, CA, United States). Data are presented as mean ± standard deviation. Next, one-way analysis of variance (ANOVA) followed by Tukey’s post hoc test was conducted for multiple statistical comparisons among cells. Then, two-way ANOVA followed by Bonferroni’s multiple comparisons test was conducted for the multiple statistical comparisons among males and females. The difference was considered significant when the *p*-value was < 0.05.

## Results

### No sex difference was detected in cell viability and IL-4 secretion

**FIGURE 2:**
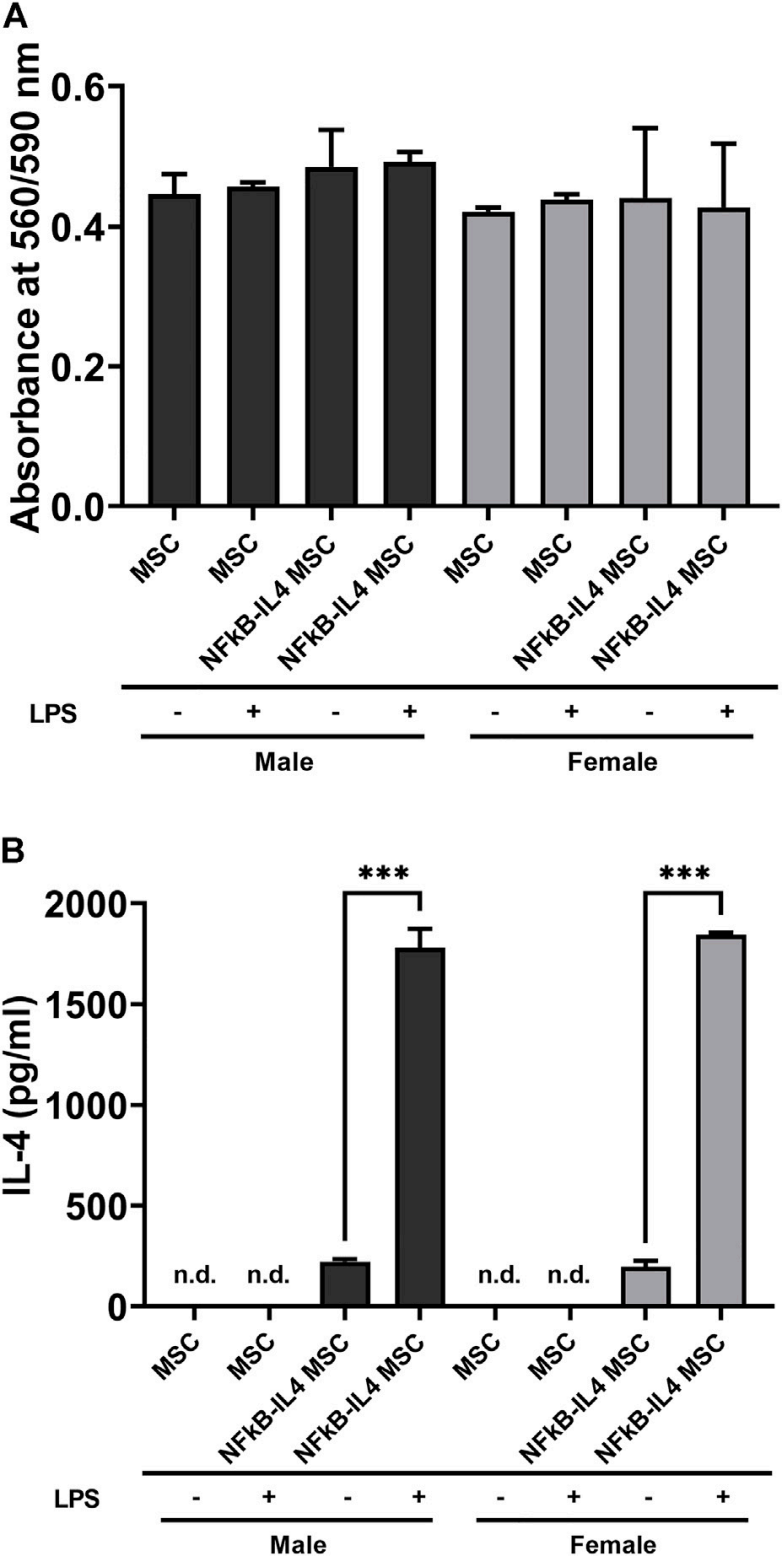
Characterization of MSCs and NFκB-IL4 MSCs from different sexes. **(A)** cell viability was compared using PrestoBlue among unaltered and NFκB-IL4 MSCs in males and females with lipopolysaccharide (LPS) challenge for 24 h. No statistical difference was found among groups. (*n* = 5 per each group for both sexes; one-way ANOVA). **(B)** IL-4 secreting detected by ELISA in the cell culture media with or without 1 ng/ml LPS treatment for 24 h. The IL-4 concentration could not be detected by in all the MSC groups in both males and females. (*n* = 3 per each group for both sexes; ^***^ *p* < 0.001, one-way ANOVA).

Unaltered and genetically modified MSCs from male and female mice demonstrated comparable cell viability, even after LPS stimulation. No significant difference was found among different cells and sexes (Figure 2A). The analysis of IL-4 secretion confirmed the success of NF-κB sensing IL-4 over-expressing lentiviral vectors infection. Levels of IL-4 were below the detectable range in all unaltered MSC; also, IL-4 secretion was significantly upregulated in the NFκB-IL-4 MSCs following LPS challenge (from 222.4 ± 12.57 to 1, 780 ± 94.1 pg/ml in males, 197.1 ± 29.91 to 1,846 ± 9.42 pg/ml in females, Figure 2B). No difference was found between males and females.

### Unaltered mesenchymal stromal cells reduced osteolysis and enhanced bone formation more than NFκB-IL-4 mesenchymal stromal cells especially in females

**FIGURE 3:**
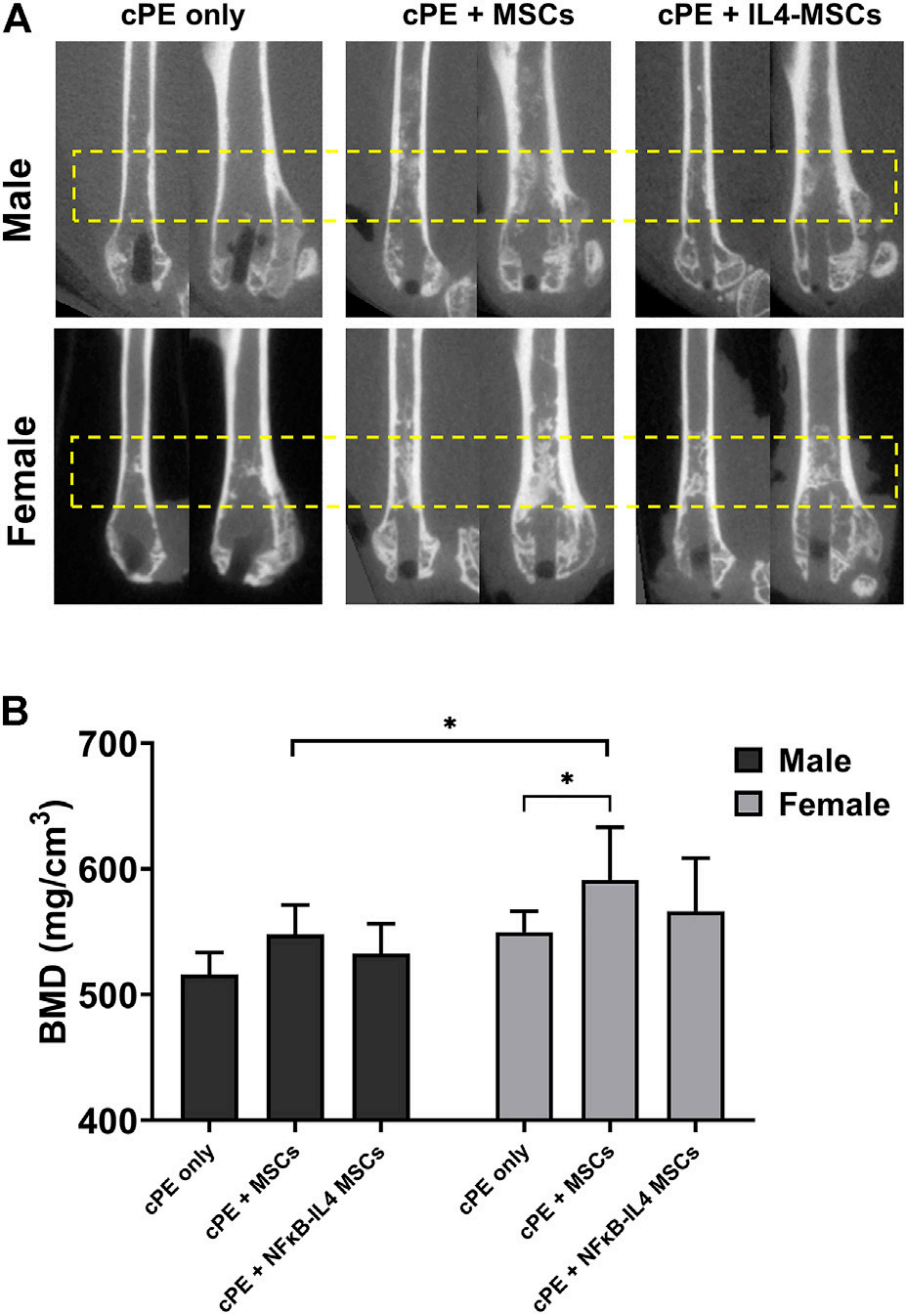
uCT examination and bone marrow density (BMD) values within the ROI for all groups at 6 weeks. **(A)** representative uCT images in sagittal and coronal planes. In both sexes, severe osteolysis appeared around the rod-shape hole in cPE only group, and it was mitigated after treatment of MSCs and NFκB-IL4 MSCs, especially in female. **(B)** quantitative assessments of BMD within the ROI. In both sexes, MSC-based therapy enhanced the BMD. MSCs group in females was significantly increased compared to sex-matched cPE only control group. Females showed significant increase compared to males in MSCs groups (Males, cPE only group: *n* = 8, cPE + MSCs group: *n* = 9, cPE + NFκB-IL4 MSCs group: *n* = 9; Females, cPE only group: *n* = 9; cPE + MSCs group: *n* = 7, cPE + NFκB-IL4 MSCs group: *n* = 6; ^*^ *p* < 0.05, Two-way ANOVA compared to sex-matched groups).

The µCT representative images in sagittal and coronal planes showed that MSC-based therapy mitigated severe osteolysis around the rod-shape hole and the reduction in cortical bone density after cPE infusion for 6 weeks (Figure 3A, yellow box). We observed an increased trend of BMD (Figure 1C) in the MSCs and NFκB-IL4 MSCs groups in both sexes (Figure 3B). BMD was significantly increased in the female MSCs group compared to the sex-matched cPE control group (*p* < 0.05). In addition, the result of female MSCs group was significantly increased compared to male MSCs groups (*p* < 0.05).

### Mesenchymal stromal cell-based therapy reduced the osteoclast-like cell number and enhanced osteoblast activity due to contaminated particles

**FIGURE 4:**
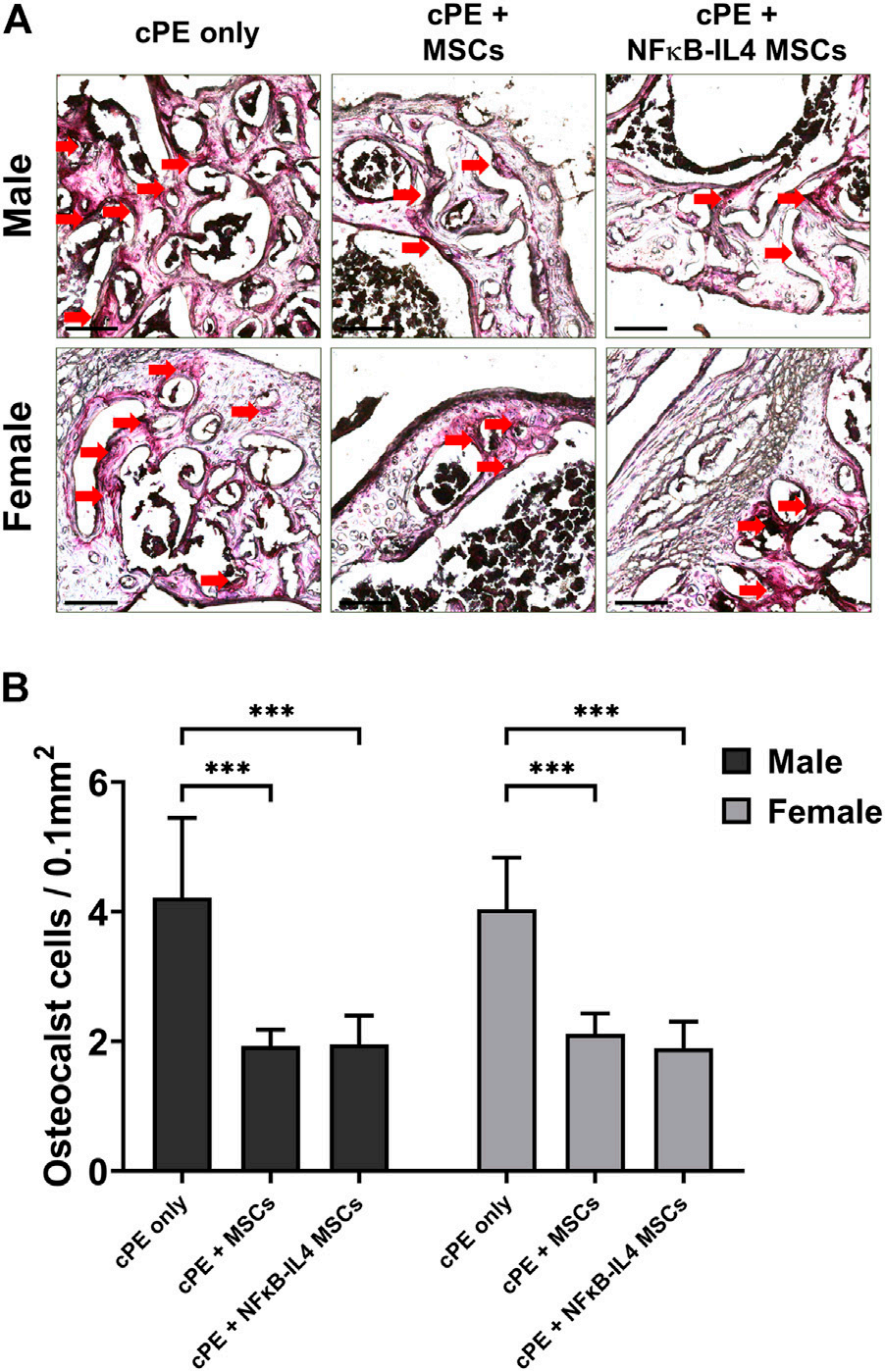
TRAP staining analysis of harvested sample at 6 weeks. **(A)** representative images of TRAP staining, red arrows point out the TRAP positive cells. Scale bar = 100 μm. **(B)** quantitative analysis of TRAP positive cells number per area. Osteoclast-like cells were significantly decreased after MSC-based therapy in both males and females. (Males, cPE only group: *n* = 7, cPE + MSCs group: *n* = 8, cPE + NFκB-IL4 MSCs group: *n* = 9; Females, cPE only group: *n* = 5, cPE + MSCs group: *n* = 7, cPE + NFκB-IL4 MSCs group: *n* = 5; ^***^ *p* < 0.001, Two-way ANOVA compared to sex-matched groups).

Numerous TRAP positive multinucleated osteoclast-like cells were identified in the cPE control group for both sexes (Figure 4A, red arrow). Both MSCs and NFκB-IL-4 over-expressing MSCs local injection resulted in a significantly decreased number of TRAP positive cells when compared to sex-matched cPE control groups (Figure 4B, *p* < 0.001). Further, no differences were observed between sexes or cell treatments.

**FIGURE 5:**
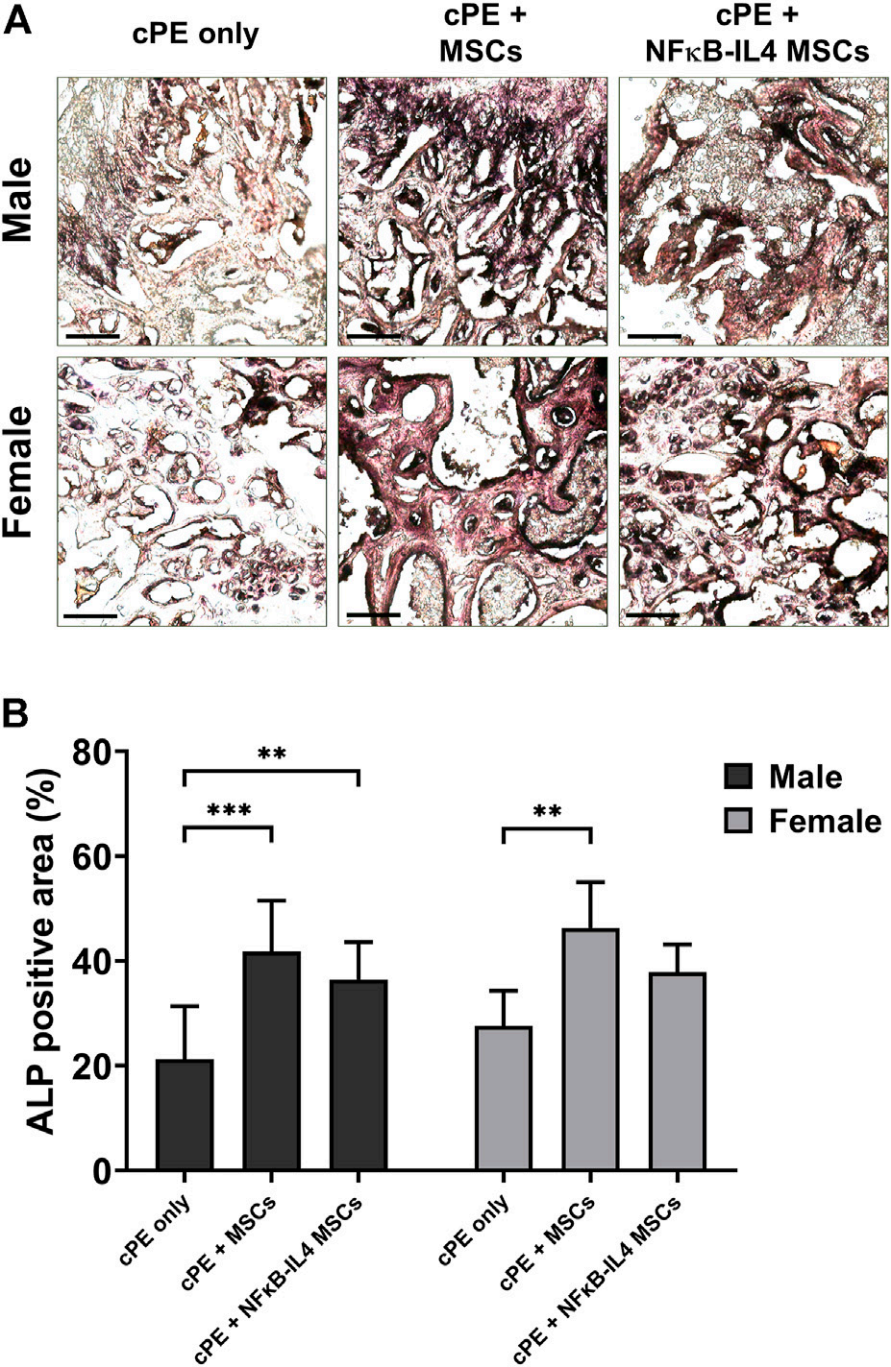
ALP staining analysis of harvested sample at 6 weeks. **(A)** representative images of ALP staining, black colored space is ALP positive area. Scale bar = 100 μm. **(B)** quantitative analysis of ALP positive area percentage. Males had significant increased ALP positive area in both MSCs and NFκB-IL4 MSCs groups. Whereas in females, only unaltered MSC group showed a statistically significant increase. (Males, cPE only group: *n* = 7, cPE + MSCs group: *n* = 8, cPE + NFκB-IL4 MSCs group: *n* = 9; Females, cPE only group: *n* = 5, cPE + MSCs group: *n* = 7, cPE + NFκB-IL4 MSCs group: *n* = 5; ^**^ *p* < 0.01, ^***^ *p* < 0.001, Two-way ANOVA compared to sex-matched groups).

Osteoblast activity, assessed by ALP staining, was significantly increased after MSCs and NFκB-IL-4 MSCs local injection in males (Figures 5A,B, *p* < 0.001 and *p* < 0.01 respectively). In females, a similar increase was observed after local injection with unaltered MSCs (Figure 5B, *p* < 0.01). NFκB-IL-4 MSCs showed a trend for enhancing osteogenesis. Overall, unaltered MSCs groups increased osteogenesis in both males and females compared to NFκB-IL-4 MSCs, and no differences were found between sexes.

### Mesenchymal stromal cell-based therapy had minimal impact on the macrophage recruitment in the presence of contaminated particles

**FIGURE 6:**
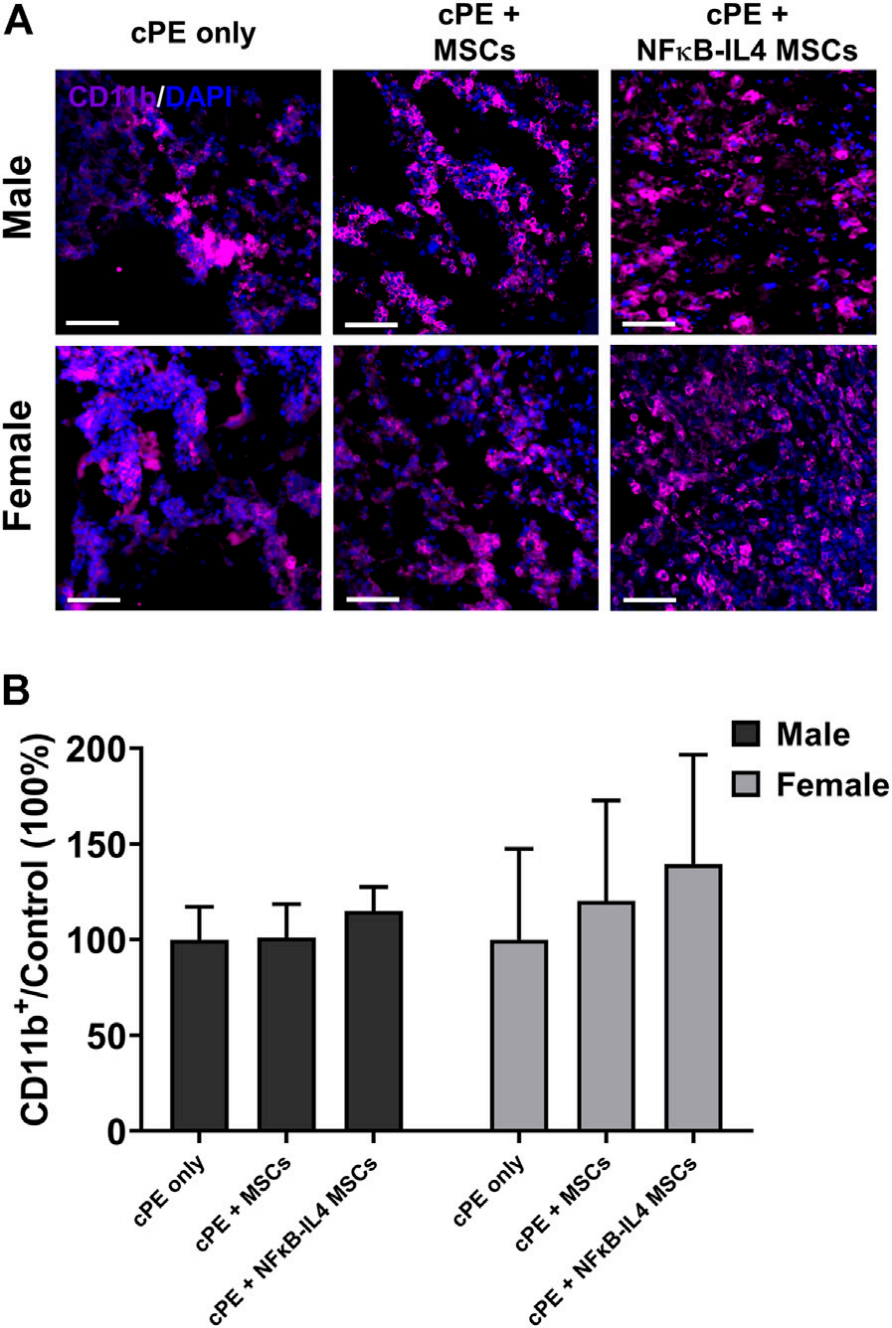
Immunofluorescent (IF) staining analysis for CD11b^+^ cells in each group. **(A)** representative images of Immunofluorescent for CD11b^+^ cells (Purple). Scale bar = 50 μm. **(B)** quantitative analysis of the ratio changes of CD11b^+^ cell compared to sex-matched control group. The CD11b^+^ cells showed an increased trend after cell treatment in MSCs and NFκB-IL4 MSCs groups, and no statistical difference was found among groups and sexes. (Males, cPE only group: *n* = 5, cPE + MSCs group: *n* = 7, cPE + NFκB-IL4 MSCs group: *n* = 5; Females, cPE only group: *n* = 5, cPE + MSCs group: n = 7, cPE + NFκB-IL4 MSCs group: *n* = 5; Two-way ANOVA compared to sex-matched groups).

Further, the innate immune response was evaluated by detecting macrophage recruitment using immunofluorescent staining. The proportional change of recruited CD11b^+^ macrophages/monocytes was not altered following either MSCs or NFκB-IL-4 MSCs local injection, compared to sex-matched control groups (Figure 6A). After MSCs based therapy, the total CD11b^+^ cells showed a trend in both MSCs and NFκB-IL4 MSCs groups, but the difference in both males and females was not statistically significant (Figure 6B)

### Female mesenchymal stromal cells had comparable immunomodulatory effects with NFκB-IL4 mesenchymal stromal cell on converting pro-inflammatory to anti-inflammatory phase

**FIGURE 7:**
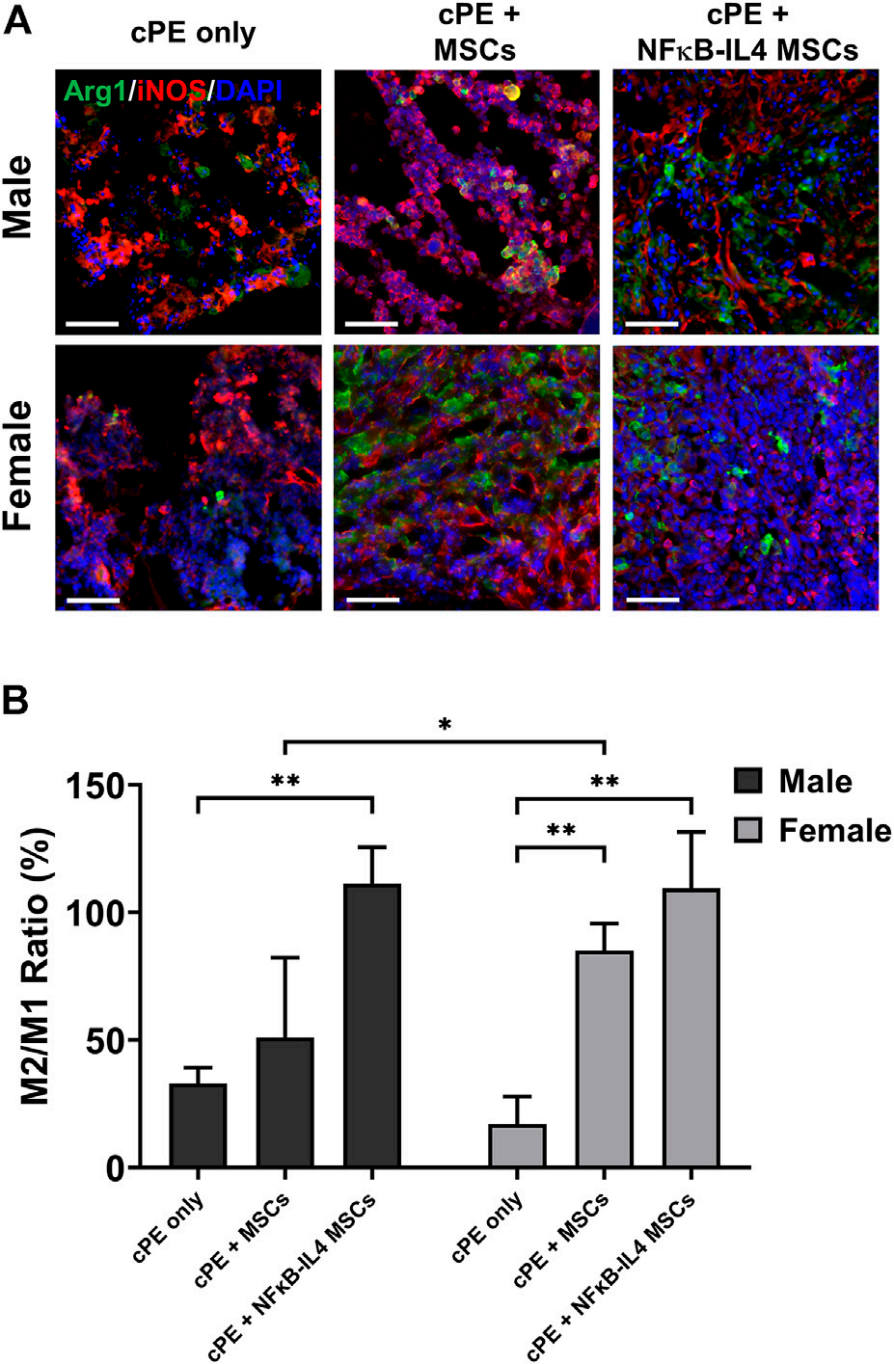
Immunofluorescent (IF) staining analysis for M1 and M2 macrophages in each group. **(A)** representative images of Immunofluorescent for M1 macrophages (iNOS^+^, Red) and M2 macrophages (Arg1^+^, Green), Scale bar = 50 μm. **(B)** quantitative analysis for cell proportion change of M2/M1 ratio. After cell treatment, males showed significantly increased M2/M1 ratio in NFκB-IL-4 MSCs group. In females, both MSCs and NFκB-IL4 MSCs groups showed significantly increased M2/M1 ratio compared to sex-matched control group. In MSC groups, females showed a significant increase compared to males. (Males, cPE only group: *n* = 5, cPE + MSCs group: *n* = 7, cPE + NFκB-IL4 MSCs group: *n* = 5; Females, cPE only group: *n* = 5, cPE + MSCs group: *n* = 7, cPE + NFκB-IL4 MSCs group: *n* = 5; ^*^ *p* < 0.05, ^**^ *p* < 0.01, Two-way ANOVA compared to sex-matched control group).

To investigate the immunomodulatory function of treatments on different sexes, the change of macrophage polarization was further analyzed. In the cPE only control group, M1 (iNOS^+^) cells were mainly distributed at the injury site; M2 (Arg1^+^) cells were rare (Figure 7A). In males, the M2/M1 ratio increased from 30% in the cPE control group to 51% in the MSCs group (ns) and to 109% in the NFκB-IL-4 MSCs group (Figure 7B, *p* < 0.01). In females, the M2/M1 ratio increased from 15% in the cPE control group to 85% in the MSCs group (*p* < 0.01) and to 107% in the NFκB-IL-4 MSCs group (Figure 7B, *p* < 0.01). In addition, the result of female MSCs group was significantly increased compared to male MSCs groups (*p* < 0.05).

## Discussion

By simulating the long-term biological processes involved in wear particle-induced periprosthetic osteolysis (Pajarinen et al., 2017), the murine femoral continuous cPE infusion model helps to understand the underlying signaling pathways and mechanisms of chronic inflammatory osteolysis. The presence of particles resulted in severe bone resorption, mainly due to the chronic inflammation through prolonged activation of NF-κB signaling (Lin et al., 2014; Liu et al., 2017). Activation of macrophages, key effector cells of innate immunity, can clear the debris released from the prosthetic articulations and help recruit mesenchymal stromal cells (MSCs) to enhance osteogenesis (Goodman et al., 2019). However, continuously accumulated cPE prevented the transition from a pro-inflammatory to an anti-inflammatory environment and promoted osteoclast differentiation and bone resorption. Furthermore, due to cytokines and other factors, the anabolic activity of pre-osteoblasts, osteoblasts, and MSCs resident in the periprosthetic microenvironment is reduced in the presence of wear particle (Granchi et al., 2005). Application of NF-κB decoy oligodeoxynucleotide (ODN), a synthesized duplex DNA that suppresses NF-κB activity through competitive binding (Lin et al., 2014; Lin et al., 2016), mitigated inflammatory osteolysis (Utsunomiya et al., 2021a). In addition, MSC-based treatments during the chronic phase at week three can effectively alleviate this phenomenon, whereas the immediate intervention impaired bone formation (Utsunomiya et al., 2021b). Furthermore, to simultaneously mitigate prolonged inflammation and enhance MSC-mediated bone formation, our established genetically modified NF-κB sensing IL-4 over-expressing MSCs can continuously release IL-4 under conditions of prolonged inflammation, promoting the polarization of M1 to M2 cells and increasing osteogenesis (Lin et al., 2017). However, various treatments in different sexes in our *in vivo* studies resulted in differences in the inflammatory response and osteogenic homeostasis.

Next, both *in vitro* and *in vivo* studies found sex differences in the function of musculoskeletal stromal cells and their response to estrogen treatment in various species (Knewtson et al., 2022). Bone is an endocrine tissue with androgen and estrogen receptors and steroid metabolizing enzymes; thus, sex hormones can demonstrate pronounced effects on bone homeostasis (Laurent et al., 2014). In recent times, osteoclast formation and callus remodeling were found to differ between sexes. The Wnt/β-catenin signaling pathway was significantly activated in male mice, resulting in faster fracture healing (Haffner-Luntzer et al., 2021). Also, sex differences using a murine long bone critical-size defect model were compared; MSC-based therapy enhanced bone bridging with increased ALP expression especially in male mice (Ueno et al., 2021). Zarei et al. demonstrated sexual dimorphism with genetic and pharmacological modulation of the alternative NF-κB pathway in bone (Zarei et al., 2019). Overall, sex differences are dynamic and are influenced by gonadal hormones, cell autonomous effects of sex chromosomes, genetics, and inflammation (Becker et al., 2005; Pietschmann et al., 2009; Mauvais-Jarvis et al., 2017). Our study compared the outcome of unaltered MSCs versus NFκB-IL4 MSCs using a murine chronic inflammatory bone loss model in males and females. At the cellular level, the gene modified MSCs continuously secreted IL-4 after NF-κB activation, and no significant difference was found in cell viability and basal secretion levels between males and females (Figures 2A,B). Mice of different sexes were treated uniformly, and MSC-based therapy demonstrated pronounced therapeutic effects in mitigating bone loss in both sexes. To be specific in females, unaltered MSCs exhibited a larger impact on the enhancement of bone formation and comparable anti-inflammatory effects with NFκB-IL4 MSCs. Also, conversely, a comparison of primary osteoblasts from neonatal calvaria showed that females demonstrated lowered ALP activity, mineralization, and related gene expression, due to the osteoblast-dominated gene *Serpina3n* (Ishida et al., 2018). Thus, in the dynamic *in vivo* environment, the overall outcome may be affected by the combined sex-related factors, including the NF-κB pathway in bone marrow cells, osteoclast activation, effects of androgen, and estrogen, etc.

Histological examination demonstrated that multinuclear osteoclasts were actively expressed at 6 weeks. Osteogenic ability was greatly reduced in the cPE only control group. The presence of cPE disrupted the homeostatic balance by activating osteoclasts and inhibiting osteoblast function, resulting in a significant decrease in the bone mass at the surgical site. In both males and females, MSC-based therapy decreased TRAP positive osteoclast number, but no significant difference was found between sexes or treatments (Figure 4B). Regarding osteogenesis, both the MSCs and NFκB-IL4 MSCs groups showed significantly increased ALP expression in males. However, in females, only the MSC group showed significant changes (Figure 5B). Our previous *in vitro* experiments showed that NFκB-IL4 MSCs co-cultured with MSCs and macrophages inhibited the early osteogenic differentiation during LPS stimulation. The expression of ALP was lower than unaltered MSCs, whereas the extracellular matrix mineralization was increased in the late stages (Lin et al., 2017; Lin et al., 2019). Estrogen deficiency by ovariectomy effectively mitigated the osteolytic response to particles using the calvarial bone model, with down-regulation of TNF-α and receptor activator of the nuclear factor kappa-B (RANKL) (Nich et al., 2011). Thus, various effects of estrogen on NF-κB pathway (Biswas et al., 2005) may result in an altered IL-4 secretion in females during the inflammatory environment. This may explain the findings in our studies in which females showed increased IL-4 associated adverse effects on MSC differentiation and osteogenesis.

Although no difference was found in osteoclast numbers between sexes after cell injection in our study, osteoclasts are key factors in sexual dimorphism of the skeletal diseases (Lorenzo, 2020). In a mouse periodontitis model, males demonstrated greater osteoclastogenesis and chemokine expression, and they were associated with higher *Nfatc1* expression (Valerio et al., 2017). Next, conversely, females showed greater bone marrow derived osteoclastogenesis than males (Mun et al., 2021). Osteoclasts are derived from circulating monocytes/macrophages and tissue resident macrophages. Most circulating inflammatory monocytes are differentiated into macrophages or preosteoclasts in the peripheral tissues and are recruited by chemokine ligand 2 (CCL2) (Shi and Pamer, 2011) and then fuse under the direction of specific cytokines (Brooks et al., 2019). Thus, targeting the macrophage-osteoclast axis may reverse the bone loss associated with wear particles (Yao et al., 2021). However, the inflammatory response is fundamental to clearing debris and bacteria, especially in the acute phase. Excessive or premature intervention may lead to undesirable effects, and effective immunomodulation at the appropriate time has important implications for final osteogenesis (Maruyama et al., 2020; Utsunomiya et al., 2021b). Next, classically activated M1 macrophages cells are highly phagocytic cells that remove pathogens and debris. To activate other macrophages to perpetuate the pro-inflammatory immune response, they trigger iNOS production and secrete other cytokines and chemokines. In contrast, the alternatively activated reparatory M2 macrophages impacts the NO production adversely with elevated levels of arginase-1 (Arg1), which attenuates the immune response and enhances cell proliferation and tissue healing (Locati et al., 2020; Kashfi et al., 2021). In our study, cPE induced chronic inflammation caused persistent osteoclast activity and recruitment of pro-inflammatory M1 dominated macrophages (Figures 6, 7). Cell treatment at week three resulted in less effects on the number of recruited macrophages but significantly promoted the polarization of M1 to M2 macrophages, especially in the NFκB-IL4 MSCs groups of both sexes. Prolonging of the pro-inflammatory phase is associated with increased osteoclasts formation (Walsh and Gravallese, 2004). On the contrary, anti-inflammatory cytokines secreted by M2 macrophages such as IL-10 and IL-4 inhibit osteoclast formation via nuclear factor of activated T cells-cytoplasmic 1 (NFATc1) (Evans and Fox, 2007). In addition, initiating the M1 to M2 transition by adding IL-4 resulted in enhanced osteogenesis including matrix mineralization, ALP activity, and osteocalcin secretion in previous *in vitro* studies (Loi et al., 2016; Lin et al., 2018). Our *in vivo* results are concordant with the *in vitro* conclusions that cell treated groups with high BMD showed largely increased M2 proportions compared with the cPE only control group. Next, interestingly, MSCs groups in females showed comparable immunomodulatory function with NFκB-IL4 MSCs, whereas males didn’t. Immune responses regarding macrophage and osteoclast activities are considered sexually dimorphic (Valerio and Kirkwood, 2018; Shepherd et al., 2020). Furthermore, intriguingly, Dou et al. demonstrated that estrogen mitigated bone loss with the protection of M2 macrophage osteoclastogenesis from RANKL via estrogen receptor *α* (ERα), both Erα selective agonists, and 17β-estradiol (E2) demonstrate comparable effects in treating osteoporotic ovariectomized (OVX) mice combined with increased M2/M1 ratio (Dou et al., 2018). Furthermore, Nathan et al. utilized LPS-induced M1 macrophages to coculture with MSCs and found that the optimal time of the M1-to-M2 transition for MSC osteogenesis is sex-dependent (Nathan et al., 2019). Thus, gene and hormone related modulations with multiple time points are necessary for further animal studies. Although the underlying mechanism of sex differences on musculoskeletal diseases remains unclear, it is now considered as an essential biological variable in preclinical studies.

Several limitations exist in this study. First, we utilized cPE only as a control group; sham surgery without cPE infusion was not conducted. Our previous study compared the outcome with or without cPE infusion (Utsunomiya et al., 2021a); thus, the present study focused on the therapeutic differences between sexes. Second, after establishing the chronic inflammatory model, cell treatments were performed at 3 weeks, and the outcome was investigated at 6 weeks after primary surgery. This time schedule aimed to mimic the long-term post-operative chronic inflammatory in clinical scenarios. Based on our previous studies, intervention at 3 weeks is more effective than immediate injection (Utsunomiya et al., 2021b). Longer experimental times and multiple time points might provide more substantial differences among different treatments or sexes. Third, to normalize potential basal functional differences at *in vitro* level, we utilized sex-matched bone marrow derived MSCs, and outcomes were investigated with uniformed treatment for each sex. Further studies are needed to investigate the effects of gender, hormonal, and genetic factors on wear particle-induced chronic inflammatory osteolysis.

In conclusion, MSC-based therapy demonstrated therapeutic effects in mitigating bone loss and simultaneously exhibited immunomodulatory functions polarizing M1 into M2 macrophages, especially with treatment of NFκB-IL4 MSCs. In females, unaltered MSCs demonstrated a greater impact enhancing bone formation but comparable anti-inflammatory effects with NFκB-IL-4 MSCs. These results demonstrated that local inflammatory bone loss can be effectively modulated via MSC-based treatments but in a sexually dimorphic manner. To conclude, the study of both sexes is essential to explore potential strategies for sex-based personalized treatments for musculoskeletal diseases.

## Data Availability

The raw data supporting the conclusions of this article will be made available by the authors, without undue reservation.
